# MOST: most-similar ligand based approach to target prediction

**DOI:** 10.1186/s12859-017-1586-z

**Published:** 2017-03-11

**Authors:** Tao Huang, Hong Mi, Cheng-yuan Lin, Ling Zhao, Linda L. D. Zhong, Feng-bin Liu, Ge Zhang, Ai-ping Lu, Zhao-xiang Bian, for MZRW Group, Shu-hai Lin, Man Zhang, Yan-hong Li, Dong-dong Hu, Chung-Wah Cheng

**Affiliations:** 1 Lab of Brain and Gut Research, School of Chinese Medicine, Hong Kong Baptist University, 7 Baptist University Road, Hong Kong, People’s Republic of China; 2 Department of Gastroenterology, the First Affiliated Hospital of Guangzhou University of Chinese Medicine, Guangzhou, 510405 People’s Republic of China; 3 YMU-HKBU Joint Laboratory of Traditional Natural Medicine, Yunnan Minzu University, Kunming, 650500 People’s Republic of China; 4 Hong Kong Chinese Medicine Clinical Study Centre, Hong Kong Baptist University, 7 Baptist University Road, Hong Kong, People’s Republic of China

**Keywords:** Explicit bioactivity, False discovery rate, Logistic regression, Mechanism-of-action target, Most-similar ligand, Target prediction

## Abstract

**Background:**

Many computational approaches have been used for target prediction, including machine learning, reverse docking, bioactivity spectra analysis, and chemical similarity searching. Recent studies have suggested that chemical similarity searching may be driven by the most-similar ligand. However, the extent of bioactivity of most-similar ligands has been oversimplified or even neglected in these studies, and this has impaired the prediction power.

**Results:**

Here we propose the **MO**st-**S**imilar ligand-based **T**arget inference approach, namely **MOST**, which uses fingerprint similarity and explicit bioactivity of the most-similar ligands to predict targets of the query compound. Performance of MOST was evaluated by using combinations of different fingerprint schemes, machine learning methods, and bioactivity representations. In sevenfold cross-validation with a benchmark Ki dataset from CHEMBL release 19 containing 61,937 bioactivity data of 173 human targets, MOST achieved high average prediction accuracy (0.95 for *pKi* ≥ 5, and 0.87 for *pKi* ≥ 6). Morgan fingerprint was shown to be slightly better than FP2. Logistic Regression and Random Forest methods performed better than Naïve Bayes. In a temporal validation, the *Ki* dataset from CHEMBL19 were used to train models and predict the bioactivity of newly deposited ligands in CHEMBL20. MOST also performed well with high accuracy (0.90 for *pKi* ≥ 5, and 0.76 for *pKi* ≥ 6), when Logistic Regression and Morgan fingerprint were employed. Furthermore, the *p* values associated with explicit bioactivity were found be a robust index for removing false positive predictions. Implicit bioactivity did not offer this capability. Finally, *p* values generated with Logistic Regression, Morgan fingerprint and explicit activity were integrated with a false discovery rate (FDR) control procedure to reduce false positives in multiple-target prediction scenario, and the success of this strategy it was demonstrated with a case of fluanisone. In the case of aloe-emodin’s laxative effect, MOST predicted that acetylcholinesterase was the mechanism-of-action target; in vivo studies validated this prediction.

**Conclusions:**

Using the MOST approach can result in highly accurate and robust target prediction. Integrated with a FDR control procedure, MOST provides a reliable framework for multiple-target inference. It has prospective applications in drug repurposing and mechanism-of-action target prediction.

**Electronic supplementary material:**

The online version of this article (doi:10.1186/s12859-017-1586-z) contains supplementary material, which is available to authorized users.

## Background

Target identification is key to understanding the mechanism-of-action of active compounds discovered from phenotypic screening or found in traditional herbal medicines. Various experimental methods, including affinity chromatography, drug affinity responsive target stability, and proteomics have been used for target identification [1]. However, these experimental approaches are laborious, expensive, and often unsuccessful. In contrast, computational target identification (also called “target prediction” or “target inference”) approaches are inexpensive, and effective. It is readily integrated with experimental validation, and can quickly narrow down potential targets to a handful of most likely candidates. A number of computational tools are available for target prediction [2]; they can be classified by algorithms into four major classes, namely, machine learning, inverse docking, bioactivity spectra analysis, and chemical similarity searching; the merits and flaws of each approach can be found elsewhere [3]. In this study, we will focus on chemical similarity searching.

Chemical similarity searching is based on the observation by medicinal chemists that structurally similar compounds usually have similar biological activities [4]. In practice, compounds are represented by two-dimensional (2D) fingerprints, and the similarity can be measured by Tanimoto coefficient (*Tc*) metrics [5]. Fingerprint-based similarity searching is widely used for target prediction. By fitting distribution of similarity between different ligand sets with extreme distribution, Keiser et al. developed the Similarity Ensemble Approach (SEA) to quantitatively calculate the correlation between different targets [6]. SEA has been successfully used in predicting new targets [7] and off-targets associated with side effects [8] of existing drugs. Similarity can also be measured by molecular shapes. For instance, Armstrong et al. proposed three-dimensional (3D) descriptors incorporating shape, chirality and charges to compare chemicals [9, 10]. One merit of molecular shape is that it can be used to detect similarity between structurally unrelated compounds, which is impossible for fingerprints. Work has been done to combine fingerprint similarity (2D) with shape similarity (3D) to improve target prediction [11, 12]. More recently, the chemical similarity network was used for target inference based on global comparison [13]. Discovering binders of a new structural class by chemical similarity searching is difficult because this approach requires high similarity with known ligands to make predictions.

Indeed, fingerprint-based similarity searching approaches have performed well in terms of accuracy and speed according to various benchmark tests of target prediction. Recently, the results of several studies imply that the most-similar counterpart of the target drives high predictive accuracy of fingerprint-based similarity searching [12, 14]. Despite these advances, the explicit bioactivity data of the most-similar ligand were oversimplified as implicit values like “active” or “inactive”. Our insight is that, if the query compound has the same similarity as two most-similar ligands belonging to targets A and B, then the probabilities of query compound being active on target A or B should not be equal. Instead, the more potent known ligand should suggest better probability. To verify this insight, we will investigate, the effects on prediction performance by explicit or implicit bioactivity in current study.

Finally, we describe a method in which we use the fingerprint similarity and explicit bioactivity data of ligands most-similar to query compounds to make inferences about their targets. We name this method “MOST”, representing “**MO**st-**S**imilar ligand-based **T**arget inference”. MOST showed high prediction accuracy with a reduced false positive rate.

## Methods

### Generation of *Ki* dataset from CHEMBL database

The bioactivity data of all human targets in CHEMBL release 19 and 20 [15] were downloaded via an in-house script written in Python. The direct binding (confidence score=”9”) bioactivity data with type “Ki” of each target were extracted and processed. Bioactivity data with unspecified concentration/activity values, unspecified concentration/activity units, unspecified references, and ambiguous operators were classified as “ineffective” and excluded. For multiple records for one target-ligand pair, if the *Ki* values were from the same publication, the smallest *Ki* (i.e. highest *pKi*) value was taken to reflect experimental optimization and/or remove unclear stereoisomer annotations [16]. After this step, if there were still multiple measurements for one target-ligand pair, which were from different publications (tested in the same or different labs), the mean *Ki* values were taken.

### Generation of sevenfold cross-validation dataset

Bioactivity data from preprocessed CHEMBL19 were used to generate cross-validation datasets. Targets which had less than 10 ligands and more than 10,000 ligands were filtered out. To make consistent comparison, only targets occurring in both CHEMBL19 and CHEMBL20 were kept. Finally, the benchmark *Ki* dataset was comprised of 173 targets annotated with 61,937 bioactivity data (Additional file 1: Table S1). These targets were covered by major drug target types, including 79 receptors, 60 enzymes, and 12 transporters (Additional file 1: Figure S1A). These targets were annotated with different number of ligands: 71 targets had 10–100 ligands; 82 targets had 100–1,000 ligands; and 20 targets had 1,000–10,000 ligands (Additional file 1: Figure S1B). The annotated ligands were further categorized into three classes by their *K*_*i*_ values. Percentages of ligands of with *K*_*i*_ values less than 1 μM, between 1- and 10 μM, and greater than 10 μM ligands were 76.7%, 17.2%, and 6.2%, respectively (Additional file 1: Figure S1C). However, such proportions were varied for specific targets (Additional file 1: Figure S1C).

15% of the ligands of each target were randomly selected to comprise the test set, and the rest were treated as the training set. This procedure was repeated seven times to make sure that the whole dataset was sampled [14]. In the training set, each ligand was selected and the most-similar ligand was generated by comparing the selected ligand with remaining ligands. In the test set, the most-similar ligand was acquired by comparing the query compound with ligand sets in the training set. To see how similar query compounds were with their most-similar counterparts, the distribution of *Tc*_most_ was determined as shown in Additional file 1: Figure S2A. A large fraction (85.4%) of query compounds had very similar (*Tc*_most_ ≥ 0.8) ligands, both in the training and test sets. Scatter plots of *pKi*_query_ vs *pKi*_most_ clearly showed that the principle, “structurally similar chemicals have similar bioactivities” [17], applies to the benchmark dataset; although there are also exceptions--some potent compounds had similar but weak binder counterparts, which is consistent with previous observations [18]. Moreover, the calculated Pearson correlation coefficient showed that structurally similar chemicals have more close bioactivities (Additional file 1: Figure S2B), suggesting that *pKi*_most_ may also be a strong predicator for the activity of a query compound.

Two threshold values of *pKi* were applied to label a ligand is “active” (represented by 1) or “inactive” (represented by 0) to a target. When *pKi* ≥ 5 was applied, 93.8% were categorized as “active”, while 6.2% were “inactive”. If *pKi* ≥ 6 was applied, 76.7% were labeled as “active”, while 23.3% were “inactive” (Additional file 1: Table S1). Inactive data were included in model training and testing since evidence has shown that negative information can improve the prediction performance [19].

### Generation of temporal validation dataset

The whole *Ki* dataset from CHEMBL19 was used as training set for temporal validation. By comparing with CHEMBL19, which was released in 2014, the newly added bioactivity data in CHEMBL20 (released in 2015) was identified and used to generate the test set. In total, there were 173 targets annotated with 3,754 *Ki* data. In this dataset, when *pKi* ≥ 5 was applied, 91.3% were categorized as “active”, while 8.7% were “inactive”. If *pKi* ≥ 6 was applied, 75.7% were labeled as “active”, while 24.3% were “inactive” (Additional file 1: Table S1).

### Calculation of fingerprint and similarity

Two fingerprint schemes were used in this study--ECFP-4-like Morgan (radius = 2) [20] calculated by RDKit [21] and FP2 calculated by OpenBabel [22]. Once fingerprints were derived, the similarity between compound pairs was calculated by Tanimoto coefficient (*Tc*) [5].

### Machine learning methods

Machine learning models, including Naïve Bayes [23], Logistic Regression [24], and Random Forests [25], were used in this study for comparison. The probability to be active (${p}_a$) as calculated by Naïve Bayes (Eq. 1) or by Logistic Regression (Eq. 2) is expressed as follows:

1 $$ \mathrm{p}\left( y\kern0.5em =\kern0.5em  active\ \Big|\  T{c}_{most},\kern0.5em  pK{i}_{most}\right)\kern0.5em =\kern0.5em \frac{p\left( T{c}_{most},\  pK{i}_{most}\Big|{}_{y= active}\right) p\left( y\kern0.5em =\kern0.5em  active\right)}{p\left( T{c}_{most}, pK{i}_{most}\right)} $$

2 $$ \mathrm{p}\left( y\kern0.5em =\kern0.5em  active\ \Big|\  T{c}_{most},\kern0.5em  pK{i}_{most}\right)\kern0.5em =\kern0.5em \frac{1}{1\kern0.5em +\kern0.5em {e}^{-\left({a}_{0+{a}_1 T{c}_{most}+{a}_2 pK{i}_{most}}\right)}} $$

where ${Tc}_{most}$ is the similarity between query compound and the most-similar ligand, while ${pKi}_{most}$ is the activity of the most-similar ligand. The sum of probabilities to be active (${p}_a$) and inactive (${p}_i$) always equal to 1. Fitting Naïve Bayes, Logistic Regression, and Random Forests models were realized by a machine learning package in scikit-learn [26].

### Workflow of MOST

**Fig. 1:**
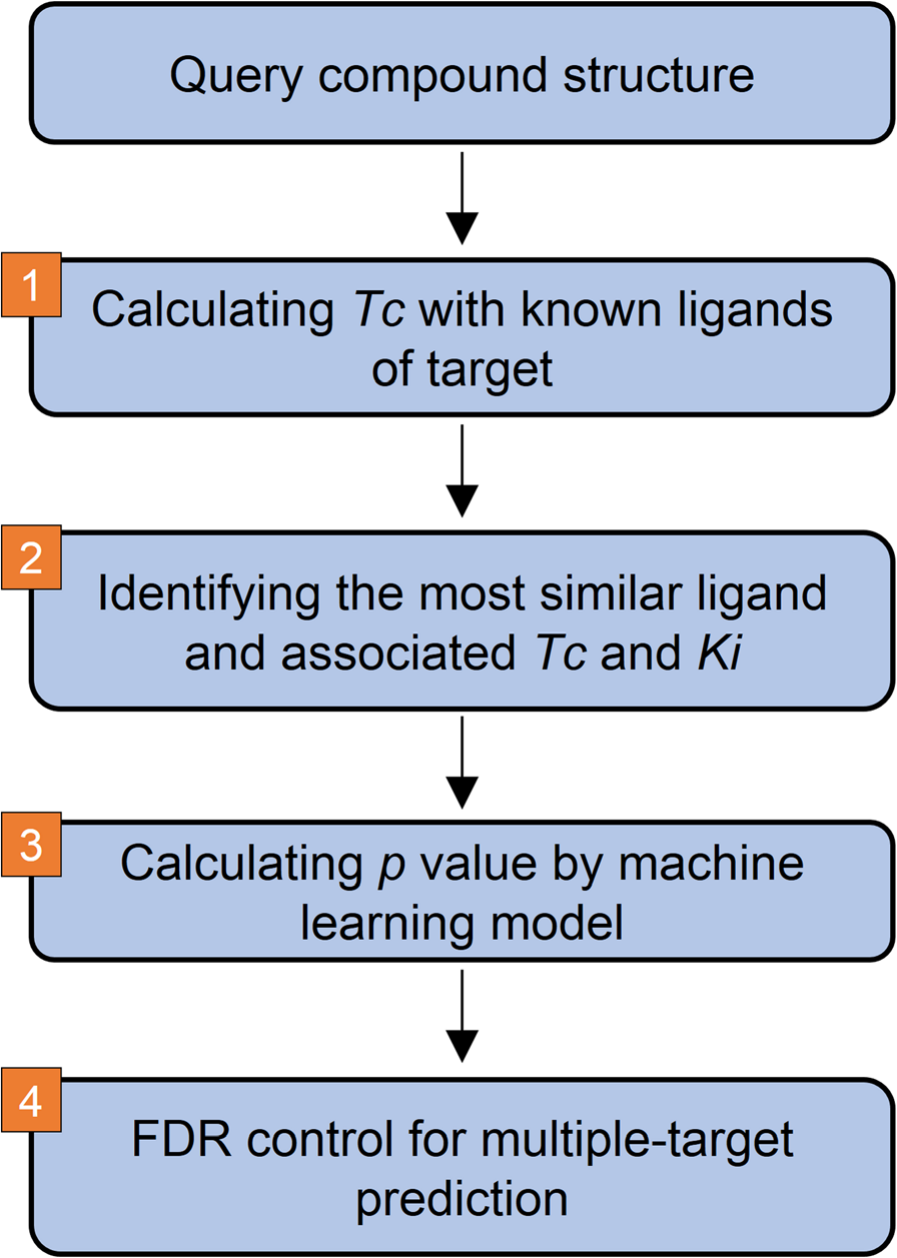
Workflow of MOST for target prediction

The workflow adopted by MOST to make predictions for a query compound with reference to a series of targets is depicted in Fig. 1. Firstly, the *Tc* values between the query compound and annotated ligands of target are calculated. Secondly, the most-similar ligand is identified by ranking the *Tc* values. Thirdly, the *Tc* and *pK*_*i*_ of the most-similar ligand (*Tc*_most_ and *pKi*_most_) are fed into a trained model to generate probabilities (*p* value) measuring how likely it is that the query compound is inactive. If explicit activity is used, the ${pKi}_{most}$ is used “as-it-is” in model training and testing. If implicit activity is used, ${pKi}_{most}\ge$5 or 6 is then simplified as 1, and ${pKi}_{most}$<5 or 6 is simplified as 0. Once the probabilities have been generated by machine learning models, if ${p}_a$>${p}_i$, the query compound is predicted to be active; otherwise, it is considered to be inactive. The probability to be inactive, ${p}_i$, is treated as *p* values in MOST for FDR control. If multiple-target predictions are made spontaneously, the FDR procedure is implemented to control the risk of false positives.

### Performance evaluation

The performance of MOST was evaluated by calculations of accuracy and Mathews Correlation Coefficient (MCC) [27, 28], according to the following equations:

3 $$ \mathrm{accuracy}\kern0.5em =\kern0.5em \frac{TP\kern0.5em +\kern0.5em  TN}{TP\kern0.5em +\kern0.5em  FN\kern0.5em +\kern0.5em  TN\kern0.5em +\kern0.5em  FP} $$

4 $$ \mathrm{M}\mathrm{C}\mathrm{C}\kern0.5em =\kern0.5em \frac{TP\kern0.5em \times \kern0.5em  TN\kern0.5em -\kern0.5em  FP\kern0.5em \times \kern0.5em  FN}{\sqrt{\left( TP\kern0.5em +\kern0.5em  FP\right)\left( TP\kern0.5em +\kern0.5em  FN\right)\left( TN\kern0.5em +\kern0.5em  FP\right)\left( TN\kern0.5em +\kern0.5em  FN\right)}} $$

where TP is true positives, TN is true negatives, FN is false negatives, and FP is false positives.

### FDR control procedure

FDR control was implemented by the ‘p_adjust’ method in the ‘stats’ library of the R package (version 3.1.2) for the Benjamini-Hochberg [29] algorithm or by the ‘q value’ method in the ‘bioconductor’ library of the R package for the Storey-Tibshirani [30] algorithm.

### Animals and fecal pellet output

Male C57BL/6 J mice weighing around 22 g (6-week old) were purchased from the Laboratory Animal Services Center, The Chinese University of Hong Kong, Hong Kong. The mice were fed with a standard rodent diet ad libitum with free access to water and were housed in rooms maintained at 22 ± 1 °C with a 12 h light/dark cycle (lights on 6:00–18:00).

Mice were randomly divided into 6 groups with 10 mice per group. Saline and aloe-emodin (3.75 mg/kg, 7.5 mg/kg, 15 mg/kg, 30 mg/kg, and 60 mg/kg) were intragastrically administrated at 9:00 a.m. All the mice were placed in individual cages without water or food. The fecal pellets for each mouse were recorded continuously for 2 h. In a parallel study, mice were randomly divided into 4 groups with 10 mice for each group. Saline and atropine (2 mg/kg and 4 mg/kg) were intragastrically administrated 20 min before aloe-emodin (15 mg/kg) treatment. Then mice were placed in individual cages without water or food, and fecal pellets for each mouse were collected within 2 h.

## Results

### Performance of MOST in sevenfold cross-validation

To evaluate the performance of MOST, three factors were evaluated in a combinational way. These factors are (1) machine learning methods (Naïve Bayes, Logistic Regression or Random Forests), (2) fingerprint schemes (Morgan or FP2), and (3) representation of bioactivity of the most-similar ligand (explicit or implicit). Accuracy and MCC under different conditions are summarized (Table 1). Firstly, Logistic Regression or Random Forests performed better than Naïve Bayes in almost all cases in terms of average accuracy and MCC; there were only marginal differences between Logistic Regression and Random Forests. Secondly, Morgan fingerprint was slightly better than FP2 in most cases. Thirdly, explicit *pKi* were as good as implicit *pKi* in terms of average accuracy. The best performance of MOST were achieved when Logistic Regression/Random Forests and Morgan fingerprint were used. For active data defined by *pKi* ≥ 5, the average accuracies were about 0.95, with MCC ranging from 0.50 to 0.59. While for active data defined by *pKi* ≥ 6, the average accuracies were about 0.87, with MCC ranging from 0.61 to 0.63.

**Table 1:** Overall performance of MOST in sevenfold cross-validation

Active data defined by	Performance						
*pKi* ≥ 5	Accuracy						
		Naïve Bayes		Logistic Regression		Random Forests	
		Morgan	FP2	Morgan	FP2	Morgan	FP2
	Explicit Ki	0.927 ± 0.003	0.929 ± 0.003	0.948 ± 0.003	0.949 ± 0.002	0.948 ± 0.001	0.946 ± 0.004
	Implicit Ki	0.939 ± 0.002	0.937 ± 0.003	0.948 ± 0.003	0.949 ± 0.003	0.950 ± 0.002	0.949 ± 0.003
	MCC						
		Naïve Bayes		Logistic Regression		Random Forests	
		Morgan	FP2	Morgan	FP2	Morgan	FP2
	Explicit Ki	0.352 ± 0.013	0.345 ± 0.017	0.495 ± 0.010	0.484 ± 0.025	0.530 ± 0.017	0.504 ± 0.032
	Implicit Ki	0.554 ± 0.008	0.543 ± 0.022	0.558 ± 0.013	0.585 ± 0.023	0.585 ± 0.014	0.560 ± 0.023
pKi ≥ 6	Accuracy						
		Naïve Bayes		Logistic Regression		Random Forests	
		Morgan	FP2	Morgan	FP2	Morgan	FP2
	Explicit Ki	0.848 ± 0.004	0.842 ± 0.002	0.866 ± 0.002	0.862 ± 0.002	0.861 ± 0.004	0.853 ± 0.004
	Implicit Ki	0.860 ± 0.004	0.855 ± 0.004	0.867 ± 0.003	0.863 ± 0.003	0.867 ± 0.004	0.862 ± 0.004
	MCC						
		Naïve Bayes		Logistic Regression		Random Forests	
		Morgan	FP2	Morgan	FP2	Morgan	FP2
	Explicit Ki	0.561 ± 0.014	0.540 ± 0.009	0.617 ± 0.009	0.602 ± 0.007	0.609 ± 0.013	0.581 ± 0.013
	Implicit Ki	0.624 ± 0.011	0.610 ± 0.612	0.632 ± 0.012	0.618 ± 0.011	0.632 ± 0.013	0.618 ± 0.012

### Performance of MOST in temporal validation

To avoid overestimation of model quality with cross-validation, we used a temporal dataset to evaluate the performance of MOST. Newly added *Ki* data in CHEMBL20 were predicted by MOST trained with *Ki* data in CHEMBL19 and the results are summarized (Table 2). In general, the performance of MOST was slightly worse in temporal validation than in cross-validation. Logistic Regression outperformed Random Forests and Naïve Bayes in this temporal validation. Morgan fingerprints were better than FP fingerprints. Similar with the cross-validation results, models with explicit- and implicit *pKi* had almost the same accuracy. The best performance was achieved when Logistic Regression and Morgan fingerprint were employed. The average accuracy was about 0.90 with MCC ranging from 0.27 to 0.29, when active data was defined by *pKi* ≥ 5, and it was about 0.76 with MCC ranging from 0.37 to 0.38, when active data was defined by *pKi* ≥ 6.

**Table 2:** Overall performance of MOST in temporal validation

Active data defined by	Performance						
*pKi* ≥ 5	Accuracy						
		Naïve Bayes		Logistic Regression		Random Forests	
		Morgan	FP2	Morgan	FP2	Morgan	FP2
	Explicit *pKi*	0.750	0.670	0.905	0.901	0.893	0.871
	Implicit *pKi*	0.741	0.696	0.896	0.894	0.896	0.897
	MCC						
		Naïve Bayes		Logistic Regression		Random Forests	
		Morgan	FP2	Morgan	FP2	Morgan	FP2
	Explicit *pKi*	0.275	0.110	0.272	0.184	0.283	0.136
	Implicit *pKi*	0.267	0.138	0.292	0.213	0.256	0.192
*pKi* ≥ 6	Accuracy						
		Naïve Bayes		Logistic Regression		Random Forests	
		Morgan	FP2	Morgan	FP2	Morgan	FP2
	Explicit *pKi*	0.633	0.554	0.755	0.736	0.724	0.709
	Implicit *pKi*	0.632	0.556	0.761	0.737	0.759	0.726
	MCC						
		Naïve Bayes		Logistic Regression		Random Forests	
		Morgan	FP2	Morgan	FP2	Morgan	FP2
	Explicit *pKi*	0.357	0.225	0.382	0.321	0.319	0.273
	Implicit *pKi*	0.334	0.212	0.370	0.307	0.381	0.300

### Benefits of using explicit *pKi*

**Fig. 2:**
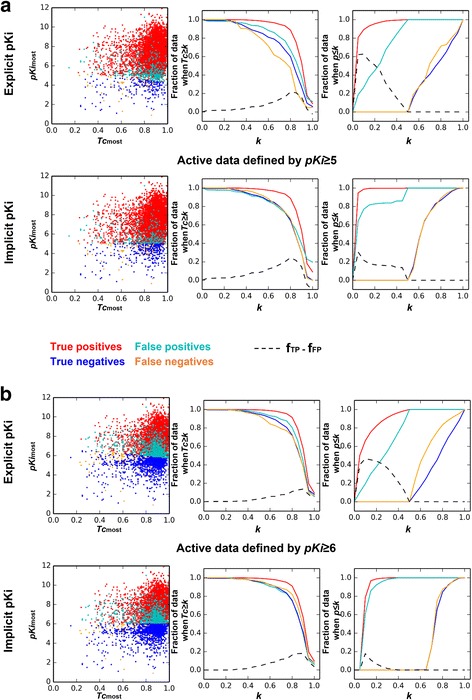
Prediction results of MOST in one dataset of sevenfold cross-validation with Logistic Regression method and Morgan fingerprint. **a** and **b**, the predicted results derived from different “active” data definition: pKi ≥ 5 and pKi ≥ 6. Results generated by using explicit and implicit Ki of most-similar ligand in model training are compared. *Left panels*, the predicted results in *Tc*_*most*_ vs *pKi*_*most*_ scatter plot. *Middle panels*, the fraction of data regarding to the increasing threshold of *Tc. Right panels*, the data fraction regarding to the decreasing threshold of *p* values. The difference between f_TP_ and f_FP_ was plotted in *black*, *dash line*. In all panels, true positives are colored *red*, while true negatives are *blue*; false positives are *cyan*, while false negatives are *orange*

To investigate the differences resulting from explicit- and implicit activity modes, one of the sevenfold cross-validation results was analyzed (Additional file 1: Table S2). Logistic Regression and Morgan fingerprint were chosen because they achieved the best performance. It was clear that more positive predictions (for both TPs and FPs) were made by explicit activity mode, compared with implicit activity mode. When all predictions were displayed in a scatter plot of *pKi*_most_ *vs Tc*_most_, many FPs were found to be predicted by most-similar ligand with weak affinity, and using explicit activity enhanced this tendency (Fig. 2a and b, left panels).

Former study suggested that setting the lower threshold (*k*) of *Tc* could reduce FPs [14]. Thus we calculated the fraction of data (f) when *Tc* ≥ *k*, while the difference between f_TP_ and f_FP_ was used as a trade-off index. Ideally, a best *k* means keeping as many TPs as possible and as few FPs as possible at the same time, which is, the maximum difference between f_TP_ and f_FP_. The f_TP_ started to fall when *Tc* ≥ *0.4*, indicating that *Tc* ≥ *0.4* was a minimum requirement for removing substantially unrelated compound pairs (Fig. 2a and b, middle panels). The difference between f_TP_ and f_FP_ reached a maximum when *Tc ≥* 0.85 in both explicit- and implicit bioactivity modes. However, the extent of difference (f_TP_-f_FP_) at this point was only about 0.2, suggesting that increasing the *Tc* threshold may not be a robust way to reduce FPs predicted by MOST.

We then wondered if setting the *p* value threshold could be a better way to reduce FPs without losing too much TPs. A decreased *p* value threshold led to rapidly decreased f_FP_ and slowly decreased f_TP_, which was only observed with explicit-, but not implicit bioactivity mode (Fig. 2a and b, right panels). Moreover, the maximum difference between f_TP_ and f_FP_ occurred when *p* ≤ 0.1: 0.60 for active data defined by *pKi* ≥ 5, or 0.40 for active data defined by *pKi* ≥ 6. These results suggested that setting the upper threshold of *p* value in explicit bioactivity mode was a better way to reduce FPs than *Tc*.

### Multiple-target prediction by MOST integrated with FDR control

**Fig. 3:**
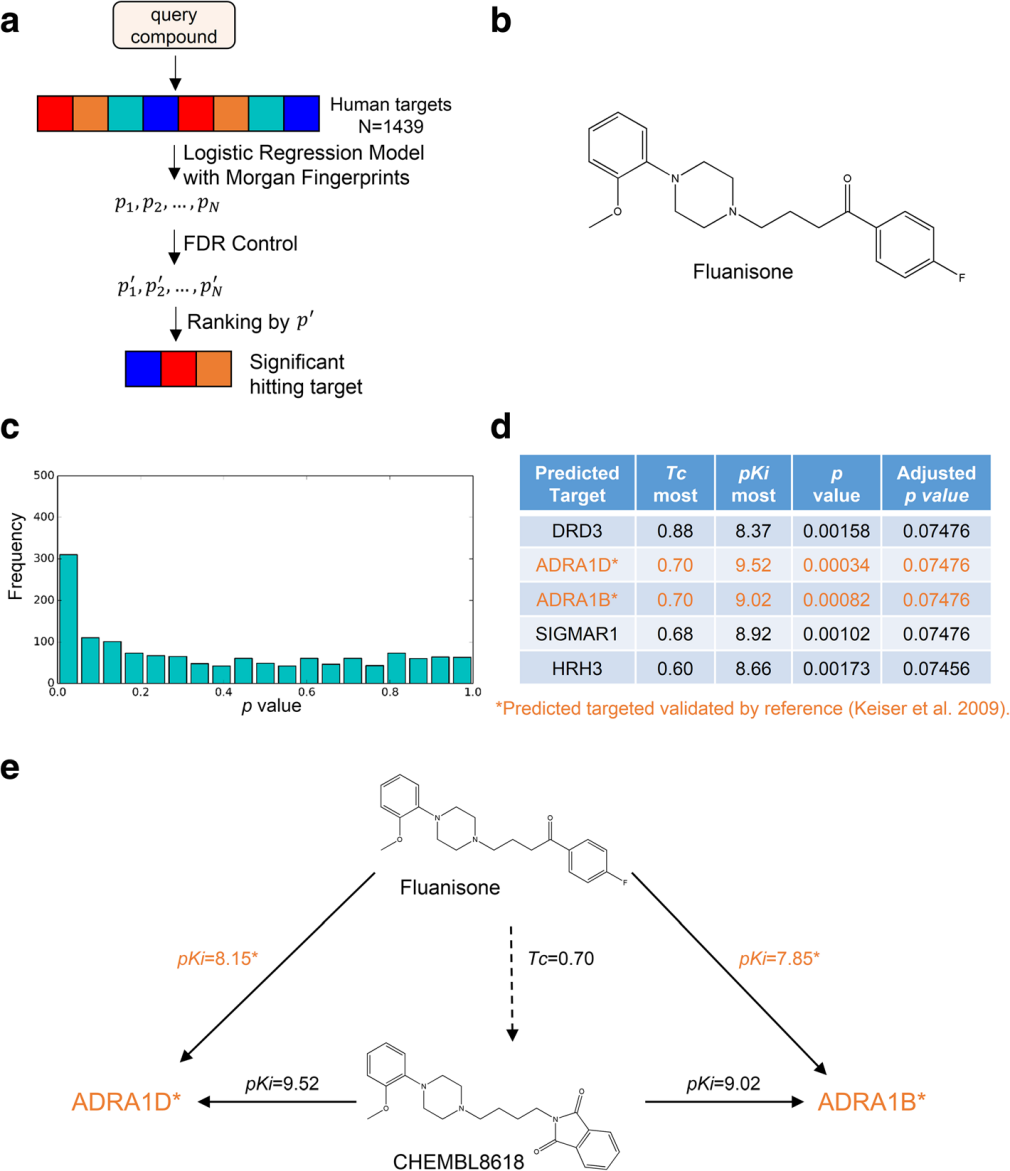
Predicting novel targets for the drug fluanisone by MOST with FDR control. **a**, scheme of integrating MOST with FDR control procedure. **b**, the structure of fluanisone. **c**, the distribution of *p* value of predicted targets, which was generated by searching fluanisone against 1,439 human targets via MOST. **d**, top 5 hits of target prediction for fluanisone. Two novel targets of fluanisone, adrenoceptor alpha 1B (ADRA1B) and adrenoceptor alpha 1D (ADRA1D), were characterized by reference (Keiser et al. [7]) but not CHEMBL database. The adjusted p values were calculated by Benjamini-Hochberg algorithm. **e**, the inference process of fluanisone novel targets by MOST. Fluanisone was found to be similar (*Tc* = 0.70) to compound CHEMBL8618, which potently acts on ADRA1B and ADRA1D. They were assigned small *p* values by MOST

One important application of MOST is to predict novel targets of known drugs, which is key to repurposing drugs and inferring side effects. In such cases, the drug will be compared with known ligands of thousands of human targets. Encouraged by the benchmark results, we evaluated MOST in multiple-target prediction, where the query compound was searched against 1,439 human targets. To avoid too many false positive predictions, FDR control procedures were introduced to correct the *p* values generated by the Logistic Regression model (Fig. 3a).

Bioactivity data of some drugs can be found in references but not the CHEMBL database, which gave us the opportunity to test if MOST can predict novel targets of such drugs in a multi-target prediction scenario. We used fluanisone, an antipsychotic and sedative drug approved for schizophrenia [31], as an example to illustrate how MOST can be used to predict novel targets for approved drugs (Fig. 3b). The *p* values of fluanisone against 1,439 human targets were calculated by Logistic Regression model with Morgan fingerprint trained by the CHEMBL 20 benchmark dataset in explicit bioactivity mode. The distribution of *p* values suggested that either the Benjamini-Hochberg or Storey-Tibshirani methods are suitable for correction (Fig. 3c). The predicted targets were ranked by adjusted *p* values and *Tc*_most_. Among the top 5 predicted targets, adrenoceptor alpha 1B (ADRA1B) and adrenoceptor alpha 1D (ADRA1D) are known human targets of fluanisone (*pK*_*i*_ equals to 7.85 and 8.15, respectively), documented in literature [7] but not in CHEMBL database. ADRA1B and ADRA1D were ranked as the 2nd and 3rd targets according to adjusted *p* values and *Tc*_most_ (Fig. 3d). Fluanisone was related to the two targets because it was quite similar (*Tc* = 0.70) to a common ligand (Fig. 3e), CHEMBL8618, which potently acts on ADRA1B (*pK*_*i*_ = 9.02) and ADRA1D (*pK*_*i*_ = 9.52). MOST assigned low *p* values to both targets (3.4E-04 and 8.2E-04) and made them top hits.

### Investigating mechanism-of-action target of aloe-emodin for laxative effect with MOST

Another important application of MOST is to predict the mechanism-of-action targets of active compounds discovered from phenotypic screening and traditional medicine. We used the laxative aloe-emodin to illustrate how MOST can be used to predict mechanism-of-action targets.

Aloe-emodin belongs to anthraquinone, a class of chemicals commonly found in the Traditional Chinese Medicine (TCM) herbs Aloe vera and Rhubarb. Aloe-emodin is found to have antibacterial, antiviral, hepatoprotective, anticancer, and anti-inflammation effects [32]. More interestingly, aloe-emodin has a laxative effect, which is in line with the traditional TCM use of Rhubarb as a laxative; however, the mechanism-of-action target of neither the herb nor aloe-emodin is not fully understood.

**Fig. 4:**
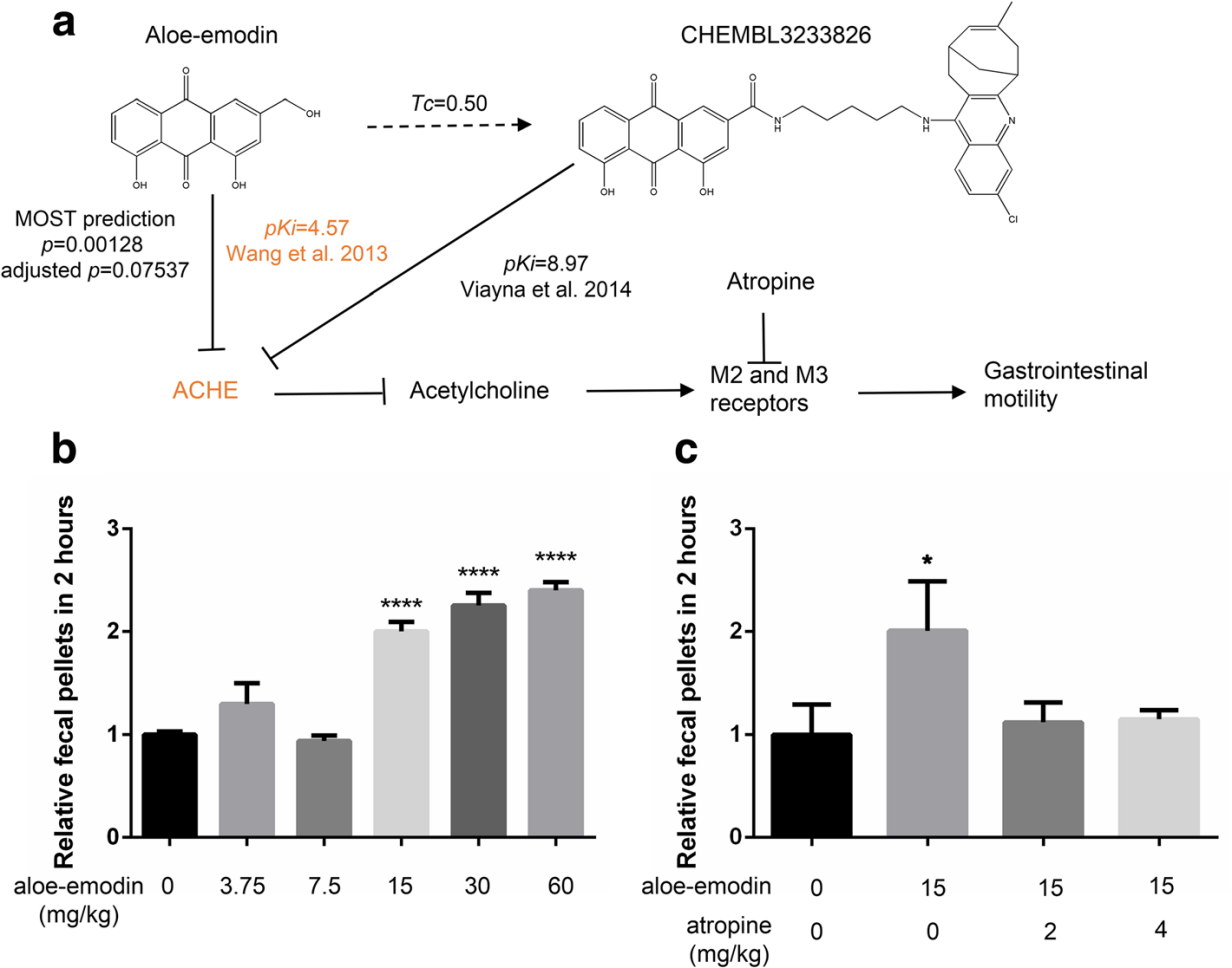
Predicting and validating the mechanism-of-action target which mediated the lataxive effect of aloe-emodin, natural product from CTM. **a**, aloe-emodin was predicted to act on acetylcholinesterase (ACHE) by MOST via the most-similar ligand, CHEMBL3233826. The IC_50_ of ACHE inhibition by aloe-emodin was reported to be 26.8 μM (Wang et al. [14]). Inhibition of ACHE results in elevating the level of acetylcholine, activating muscarinic receptors (M2 and M3), and enhancing the gastrointestinal motility. **b**, aloe-emodin dose-dependently stimulated the fecal pellets in mice. **c**, the stimulative effect of aloe-emodin was abolished by muscarinic receptors antagonist, atropine. For each group, the relative fecal pellets in 2 h were compared with the control group, and tested by unpaired *t*-test in Prism 6 (n = 10; ****, p < 0.0001; *, p < 0.05). All data in **b** and **c** are presented in Mean ± S.E.M

By using MOST, we searched aloe-emodin against 1,439 human targets, and found that acetylcholinesterase (ACHE) was the top target (Fig. 4a). Aloe-emodin was similar (*Tc* = 0.50) to CHEMBL3233826, the rhein-derived compound which potently (*pKi* = 8.97) inhibits ACHE [33]. Actually, aloe-emodin was shown to inhibit ACHE with *pKi* = 4.57 in an early study [34]. ACHE is the enzyme involved in the rapid hydrolysis of acetylcholine in numerous cholinergic pathways. Inhibition of ACHE results in accumulation of acetylcholine and hyperstimulation of the gastrointestinal smooth muscles via muscarinic M2 and M3 receptors [35, 36]. Indeed, one of the ACHE inhibitors, acotiamide is approved as a prokinetic agent for treating functional dyspepsia [37].

Given these facts, we tested whether the laxative effect of aloe-emodin is mediated by the acetylcholine signaling pathway. In C57 mice, aloe-emodin significantly increased the production of fecal pellets within 2 h after treatment with doses of 15-, 30-, and 60 mg/kg (Fig. 4b). The intragastric pre-treatment of atropine (2- and 4 mg/kg), given 20 min beforehand, totally abolished the stimulatory effect of aloe-emodin (15 mg/kg) on mice fecal pellet output (Fig. 4c). These results suggest that the cholinerigic pathway is involved in the laxative effect of aloe-emodin on mice colonic motility.

## Discussion

Utilizing the fact that similar compounds have similar bioactivity profiles [17, 38], similarity searching is one of the most simple, but robust, approaches to ligand-based target prediction. The earliest example was PASS, in which chemicals were represented by MNAs (multilevel neighborhoods of atoms) descriptors [39]. A Bayesian model was employed to train 31,000 bioactive substances, and the biological activity spectra, including 319 types of pharmacological effects, action mechanisms and toxicities, were predicted in the form of probabilities. By analyzing the similarity between ligand sets of various targets, Keiser et al. proposed SEA, which uses ensemble similarity to make target prediction [6]. In SEA, the relationship of a compound with a biological target is determined by calculating the sum of fingerprint similarities of known ligands annotated with that target, and *Tc* ≥ 0.57 was used to remove substantially unrelated ligands. The prediction significance was accessed by “BLAST-like” Z-score and *p* values according to a pre-fitted probability distribution in SEA. Unlike SEA, the mean of similarity to multiple ligands of target is utilized with multi-category Bayes classifiers to improve the performance of ligand-based target inference [40, 41]. However, it seemed not to be necessary to use all ligand information, whether in sum or mean, when relating compound with target annotated with multiple ligands. It was firstly demonstrated with MCM algorithm [42], and later proved in TargetHunter, that performance of similarity searching can be dominated by top similar ligands [14]. Except Bayes classifiers, other machine learning methods such as Support Vector Machines (SVMs) [43], Logistic Regression [12, 44], and Random Forests [28], were also employed for task of target prediction.

In the current study, we demonstrated that, solely using the information of most-similar ligands, MOST achieves high prediction accuracy. We also investigated the effects of using explicit bioactivity of most-similar ligand, which has usually been oversimplified as category values (implicit bioactivity) in previous similarity searching approaches. There was only little difference in prediction accuracy between MOST using explicit- and implicit bioactivities. In both cases, a large fraction of FPs were found to result from most-similar ligand with high *Tc* (>0.8) but low *pKi* values. This is an important finding; it suggests that simply using a *Tc* threshold cannot reduce the major part of FPs. In such case, using explicit bioactivity of most-similar ligand provides a significant advantage over implicit bioactivity because, in explicit bioactivity mode, more potent ligands will generate better probability, while less potent ligands will give worse. That’s why when *p* value threshold was applied, a large fraction of FPs were removed, while most TPs remained.

One limitation of the current study was the unbalanced training dataset—that is, the dataset included more “active” data and less “inactive data”. Since we extracted all *Ki* data with well-annotated references from the CHEMBL database, it seemed that researchers may be more likely to report positive, rather than negative results in their publications. The effects of skewed dataset were evaluated by MCC, which is more suitable for unbalanced datasets. If more negative data from other sources is included, the prediction performance can be further improved, as demonstrated in the work by Mervin *et al* [19].

We also demonstrated the application of MOST in the ‘real-world’ case of aloe-emodin. Considering there is large unmet need to elucidate the mechanism-of-action targets of traditional medicine, MOST can be optimized for specific application domains, like biological function networks or disease pathways, which are influenced by traditional medicine therapies [45].

## Conclusions

Taken together, the results reported here show that MOST is a highly accurate approach to predicting targets. Logistic Regression and Random Forests learning methods performed better than Naïve Bayes in cross-validation, while Logistic Regression outperformed the other two in temporal validation. MOST has more power to detect more positive results with explicit activity. The *p* value, rather than *Tc*, is a robust way to filter out false positives. Integrated with the FDR control procedure, MOST provides a reliable framework to predict novel targets for known drugs and to predict the mechanism-of-action targets for active compounds from traditional medicines. These capabilities have been demonstrated via the examples of fluanisone and aloe-emodin. Success of MOST as reported here may have been partly because many query compounds had highly similar counterparts in datasets used in this study. If the query compounds are from a very different structural class than the ones in training dataset, MOST may be less accurate. Despite this potential limitation, MOST is a powerful approach to relating pharmaceuticals with their potential targets.

## Additional file

Additional file 1: Figure S1. Benchmark Ki dataset for evaluating the performance of MOST generated from CHEMBL19. **Figure S2.** Distribution of *Tc*_most_ and correlation between *pKi*_most_ and *pKi*_query_ in the training and test sets. **Table S1.** Statistics of Ki datasets generated from CHEMBL19 and CHEMBL20. **Table S2.** Prediction results of MOST with Logistic Regression method and Morgan fingerprint in sevenfold cross-validation. (DOCX 583 kb)

## Abbreviations

ACHE: acetylcholinesterase
ADRA1B: adrenoceptor alpha 1B
ADRA1D: adrenoceptor alpha 1D
FDR: false discovery rate
FN: false negatives
FP: false positives
MCC: Mathews Correlation Coefficient
*Tc*: Tanimoto coefficient
TCM: Traditional Chinese Medicine
TN: true negatives
TP: true positives
