# Supplementary material for: MOST: most-similar ligand based approach to target prediction
Source: BMC Bioinformatics. 2017 Mar 11;18:165. doi: 10.1186/s12859-017-1586-z (PMC5346209; doi:10.1186/s12859-017-1586-z)
Supplement: Additional file 1: Figure S1. — Benchmark Ki dataset for evaluating the performance of MOST generated from CHEMBL19. Figure S2. Distribution of Tc most and correlation between pKi most and pKi query in the training and test sets. Table S1. Statistics of Ki datasets generated from CHEMBL19 and CHEMBL20. Table S2. Prediction results of MOST with Logistic Regression method and Morgan fingerprint in sevenfold cross-validation. (DOCX 583 kb) [file 12859_2017_1586_MOESM1_ESM.docx]

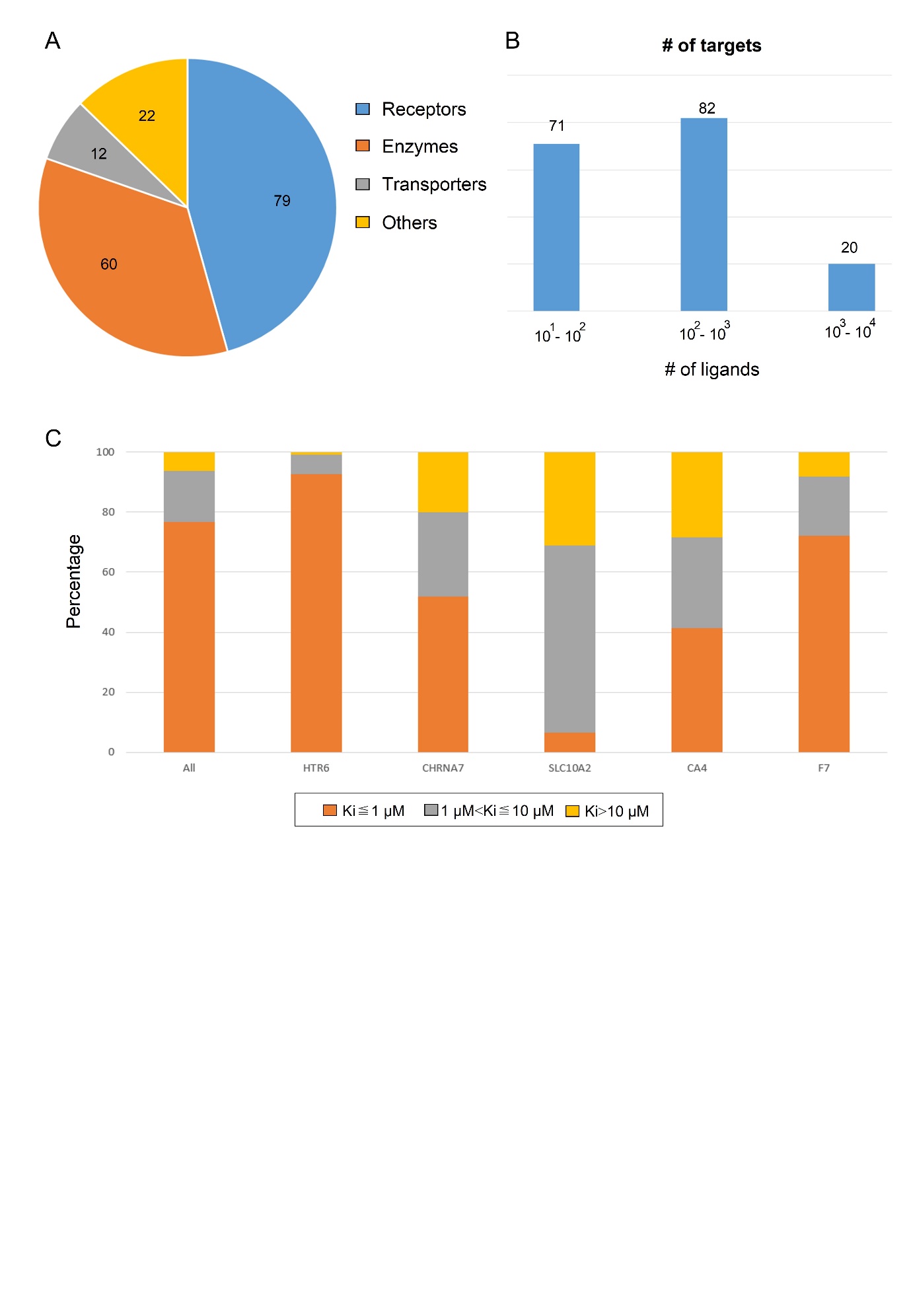


**Figure S1.** Benchmark Ki dataset for evaluating the performance of MOST generated from CHEMBL19. A, the drug target types in benchmark Ki dataset. The number of targets belonged to different types was labeled. B, the number of targets which have certain number of annotated ligands. C, the percentage of ligands classified by activity ranges in all targets and selected examples.


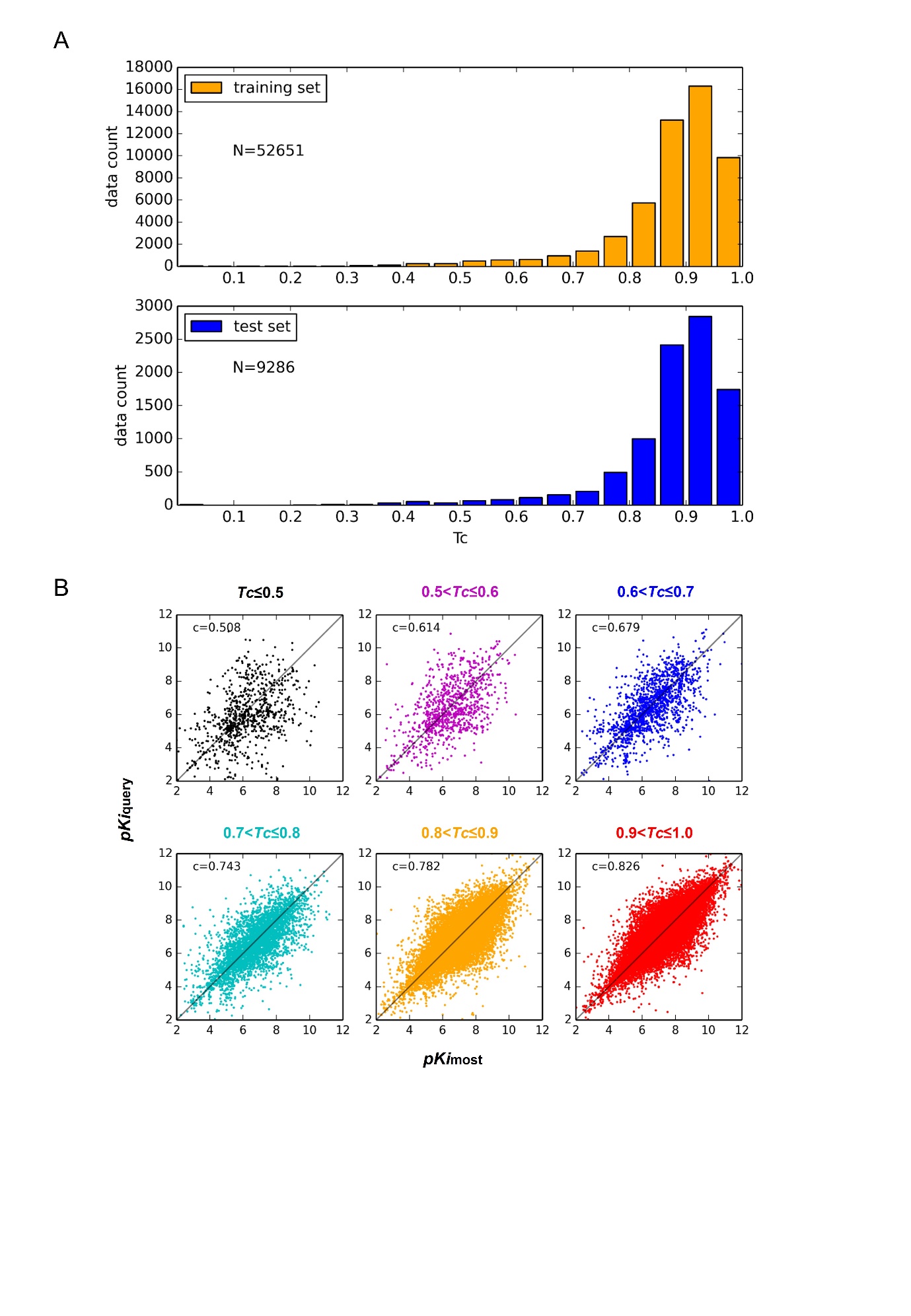


**Figure S2**. Distribution of *Tc_most_* and correlation between *pKi_most_* and *pKi_query_* in the training and test sets. A, the distribution of *Tc* similarity with the most-similar ligands (*Tc_most_*) in the training and test sets. B, the correlation between bioactivity of query compounds (*pKi_query_*) and most-similar ligands (*pKi_most_*) in the benchmark dataset. Similar compound pairs have similar activity. More similar compound pairs have stronger correlation of their activities.

**Table S1. Statistics of Ki datasets generated from CHEMBL19 and CHEMBL20.**

|  | **CHEMBL19** | **CHEMBL20** | **Incrementally deposited data in CHEMBL20 compared with CHEMBL 19** |
| --- | --- | --- | --- |
| # of unique targets | 173 | 173 | Not applicable |
| # of Ki data | 61,937 | 65,522 | 3,754 |
| # of modestly and highly active data  (*pKi*≥5 or *Ki*≦10 μM) | 58,123 (93.8%) | 61,420 (93.7%) | 3,427 (91.3%) |
| # of highly active data  (*pKi*≥6 or *Ki*≦1 μM) | 47,492 (76.7%) | 50,160 (76.5%) | 2,842 (75.7%) |

**Table S2. Prediction results of MOST with Logistic Regression method and Morgan fingerprint in sevenfold cross-validation**

| **Active data defined by** | **Dataset** | **# of total data in training set** | **# of active data in training set** | **# of total data in test set** | **# of active data in test set** | ***pKi* for prediction** | **# of TP*** | **# of FN*** | **# of TN*** | **# of FP*** |
| --- | --- | --- | --- | --- | --- | --- | --- | --- | --- | --- |
| *pKi*≥5 | 1 | 52651 | 49116 | 9286 | 8615 | *Explicit* | 8552 | 63 | 225 | 446 |
|  |  |  |  |  |  | *Implicit* | 8434 | 181 | 351 | 320 |
|  | 2 | 52651 | 49092 | 9286 | 8639 | *Explicit* | 8565 | 74 | 226 | 421 |
|  |  |  |  |  |  | *Implicit* | 8457 | 182 | 353 | 294 |
|  | 3 | 52651 | 49067 | 9286 | 8664 | *Explicit* | 8593 | 71 | 232 | 390 |
|  |  |  |  |  |  | *Implicit* | 8481 | 183 | 337 | 285 |
|  | 4 | 52651 | 49091 | 9286 | 8640 | *Explicit* | 8585 | 55 | 210 | 436 |
|  |  |  |  |  |  | *Implicit* | 8448 | 192 | 330 | 316 |
|  | 5 | 52651 | 49070 | 9286 | 8661 | *Explicit* | 8594 | 67 | 213 | 412 |
|  |  |  |  |  |  | *Implicit* | 8484 | 177 | 332 | 293 |
|  | 6 | 52651 | 49038 | 9286 | 8693 | *Explicit* | 8636 | 57 | 209 | 384 |
|  |  |  |  |  |  | *Implicit* | 8525 | 168 | 317 | 276 |
|  | 7 | 52651 | 49094 | 9286 | 8637 | *Explicit* | 8566 | 71 | 225 | 424 |
|  |  |  |  |  |  | *Implicit* | 8451 | 186 | 330 | 319 |
| *pKi*≥6 | 1 | 52651 | 39893 | 9286 | 6982 | *Explicit* | 6625 | 357 | 1432 | 872 |
|  |  |  |  |  |  | *Implicit* | 6503 | 479 | 1577 | 727 |
|  | 2 | 52651 | 39884 | 9286 | 6991 | *Explicit* | 6568 | 423 | 1481 | 814 |
|  |  |  |  |  |  | *Implicit* | 6427 | 564 | 1626 | 669 |
|  | 3 | 52651 | 39819 | 9286 | 7056 | *Explicit* | 6636 | 420 | 1432 | 798 |
|  |  |  |  |  |  | *Implicit* | 6503 | 553 | 1571 | 659 |
|  | 4 | 52651 | 39872 | 9286 | 7003 | *Explicit* | 6609 | 394 | 1435 | 848 |
|  |  |  |  |  |  | *Implicit* | 6472 | 531 | 1607 | 676 |
|  | 5 | 52651 | 39863 | 9286 | 7012 | *Explicit* | 6572 | 440 | 1439 | 835 |
|  |  |  |  |  |  | *Implicit* | 6437 | 575 | 1572 | 702 |
|  | 6 | 52651 | 39801 | 9286 | 7074 | *Explicit* | 6686 | 388 | 1331 | 881 |
|  |  |  |  |  |  | *Implicit* | 6534 | 540 | 1473 | 739 |
|  | 7 | 52651 | 39896 | 9286 | 6979 | *Explicit* | 6550 | 429 | 1468 | 839 |
|  |  |  |  |  |  | *Implicit* | 6447 | 532 | 1600 | 707 |

TP, true positives; FN, false negatives; TN, true negatives; FP, false positives.
